# Metabolomic datasets of an apple progeny carrying resistance quantitative trait loci to apple scab before or after inoculation of the pathogen *Venturia inaequalis*

**DOI:** 10.1016/j.dib.2025.111566

**Published:** 2025-04-16

**Authors:** Romane Lapous, Florent Magot, Romain Larbat, Caroline Denancé, Christian Cattanéo, Hélène Muranty, Charles-Eric Durel, Julie Ferreira de Carvalho

**Affiliations:** a University of Angers, Institut Agro, INRAE, IRHS, SFR QUASAV, F-49000 Angers, France; b Université de Lorraine, LAE, INRAE, F-54000 Nancy, France

**Keywords:** *Malus domestica*, QTL, Genetic resistance mechanisms, Biotic interactions, Metabolomics

## Abstract

Phytosanitary treatments are massively used in orchards to fight apple scab, a disease caused by the fungus *Venturia inaequalis* (*Vi*). To reduce these treatments, resistant varieties are largely deployed but their effectiveness can decrease over time. The combination of complementary molecular mechanisms within new varieties could enhance the durability of genetic resistance however, the underlying resistance mechanisms remain poorly understood. An apple pseudo-F1 progeny was previously widely investigated for its quantitative trait loci (QTL) controlling resistance to scab and at least three of them seem to act complementarily; notably, one of them is specific to some *Vi* isolates while the others have a broader spectra of action. The aim of this approach is to better understand the underlying molecular mechanisms and metabolites associated with resistance alleles by exploring apple leaf specialized metabolism. A total of three experiments was conducted: one experiment included non-inoculated leaves whereas in the two other experiments, leaf samples were collected five days after inoculation with two different *Vi* isolates, including one known to overcome one QTL. Metabolic content was extracted in aqueous methanol before performing an untargeted metabolomic analysis using an Orbitrap IDX^TM^ mass spectrometer, allowing high-resolution mass spectrometry (HRMS) detection. This approach without *a priori* enables the detection of potentially new chemical families involved in resistance to apple scab. The current data article includes 1) the protocol of plant sample production with a table summarizing key elements of the experimental designs, 2) overview of the raw metabolomic profiles from all three experiments and 3) assessment of metabolic feature reproducibility between replicates in each dataset through Principal Component Analysis. The raw data files are available on the recherche.data.gouv repository (10.57745/XJBD8V). These datasets are valuable resources to further investigate the molecular mechanisms underlying genetic resistance to apple scab, with a focus on specialized metabolism. In the long term, it should improve apple breeding strategies by informing on how to combine appropriate genetic and biochemical factors in new varieties to ensure a more durable resistance.

Specifications Table

**Table:** 

Subject	Agricultural Sciences: Horticulture
Specific subject area	Metabolomics and genetic resistance to biotic stress using two distinct isolates of a pathogen.
Type of data	Tables/figures (.pptx), raw metabolomic profiles of samples from all three experiments including sample IDs, experimental information and quantification of all metabolic features (.csv) and analytic workflow for metabolomic analyses (.txt)
Data collection	Data were obtained from leaves of grafted apple trees carrying QTL for apple scab resistance and artificially inoculated –or not- with two isolates of *Vi* in three independent experiments*.* Metabolic content of leaves was extracted in aqueous methanol and analysed using a Vanquish UHPLC system equipped with a XB-C18 Kinetex column. HRMS^1^ detection was performed on an Orbitrap IDX^TM^ mass spectrometer with ESI in positive and negative modes. HRMS^2^ analysis was performed on pooled-sample vial using DDA mode and the AcquireX data acquisition workflow (ThermoFisher).
Data source location	Institution: Research Institute for Horticulture and Seeds (IRHS), University of Angers, Institut Agro, INRAE City/Town/Region: 49071 Beaucouzé Country: France Latitude and longitude for collected samples/data: Not available
Data accessibility	Repository name: Entrepôt-Catalogue Recherche Data Gouv (https://entrepot.recherche.data.gouv.fr/) Data identification number: doi: 10.57745/XJBD8V Direct URL to data: 10.57745/XJBD8V
Related research article	None

## 1 Value of the Data

• These metabolomic datasets can be used to identify novel metabolic pathways associated to resistance QTL (rQTL), either constitutively expressed or in response to two isolates of *Vi*.
• Knowing the molecular mechanisms underlying rQTL can assist further research in apple breeding by pyramiding sources of resistance which complement each other, in order to improve long-term resistance. Notably, it will provide information needed for breeders to identify interesting genotypes to integrate in breeding schemes.
• These data can be analysed with univariate, bivariate and multivariate methods. It can also be analysed by combining genotypic data of the progeny in order to determine the genetic architecture of specialized metabolism.
• By employing an untargeted approach, unexplored chemical families may be retrieved from these datasets. To the best of our knowledge, this is one of the first datasets allowing the study of genetic determinism of untargeted metabolic features after infection of apple trees with scab strains.

## 2 Background

Diversifying the molecular mechanisms associated to resistance in new varieties by pyramiding both qualitative and quantitative resistance genes might enhance the durability of genetic resistance [1]. While major-effect R genes are usually associated with pathogen perception [2], functions of resistance quantitative trait loci (rQTL) remain poorly understood. Previous studies highlighted the complementary effects of three rQTL to apple scab [3,4], segregating in a biparental population known as ‘TxF progeny’ [3]. The rQTL ‘qT1’, coming from the hybrid TN10-8, is considered as a ‘major-gene like’ rQTL due to its isolate-specificity and strong resistance symptom induction. In addition to these characteristics, qT1 co-localized with the major *Rvi6* gene, suggesting that it could be an allele or paralog of *Rvi6* with quantitative effect [[5], [6], [7]]. On the other hand, ‘qF11’ and ‘qF17’ (from the cultivar ‘Fiesta’) interact together synergistically, which can be interpreted as the complementary action of two genes involved in a common metabolic pathway [3,8]. To further understand the molecular mechanisms underlying these QTLs, we studied the metabolome of the ‘TxF progeny’ before and after inoculation with two isolates, including one known to overcome ‘qT1’. These datasets will allow identifying specific metabolites associated with resistance to apple scab for each of these QTL.

## 3 Data Description

Metabolomic datasets were acquired from three distinct experiments (‘No inoc.’, ‘Vi-B04’, ‘Vi-BCZ14’) that will be briefly presented in this section (for more details, see EXPERIMENTAL DESIGN, MATERIALS AND METHODS). In each experiment, leaves were collected on grafted apple trees varying for their resistance to apple scab that were artificially inoculated –or not- with two isolates of *Vi*. Experimental design did not include randomization for the ‘No inoc.’ experiment whereas a block-design organization was set up for ‘Vi-B04’ and ‘Vi-BCZ14’ experiments. Metabolic content was analysed after aqueous methanol extraction using an Ultra High Performance Liquid Chromatography (UHPLC) system coupled with High-Resolution Mass Spectrometry (HRMS) detection.

The dataset contains three files, one for each experiment. Each metabolic feature is unique because it corresponds to a specific mass-to-charge ratio (*m*/*z*) measured by HRMS, at a given retention time depending on UHPLC conditions. These two metrics were coupled to attribute to each feature a unique identifier: as an example, a metabolic feature with a *m*/*z* value of 275.09129 and a time retention of 19.951 minutes was renamed as “M275.09129_T19.951”. The same process of metabolic extraction and analysis workflow was applied for all experiments and led to the identification of 1,576, 2,102 and 2,955 metabolic features in the ‘No inoc.’, ‘Vi-B04’ and ‘Vi-BCZ14’ experiments, respectively. Each line represents one sample while metabolic features are indicated in columns. An additional column defines the replicate or copy number within the block design for ‘Vi-B04’ and ‘Vi-BCZ14’ experiments. The quantitative variable for each metabolic feature corresponds to the surface area under the chromatogram curve, which is proportional to ionization properties of metabolic features and to their relative abundance in samples.

**Fig. 1:**
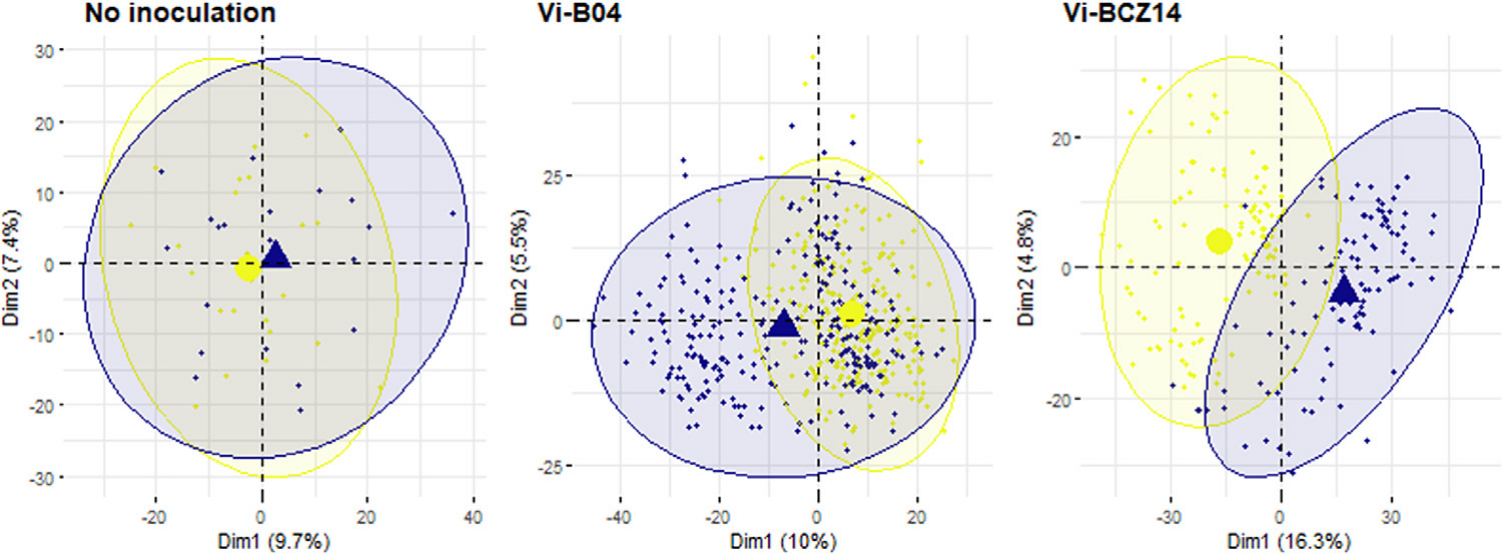
Assessment of metabolic feature reproducibility between replicates in the three experiments. The two first components of Principal Component Analysis are shown, with samples coloured according to the replicate (*No inoculation*) or block (*Vi-B04/Vi-BCZ14*) number.

Principal Components Analysis (PCA) was generated after scaling metabolic features to the standard deviation for the three conditions (Fig. 1). The first components of ‘No inoc.’, ‘Vi-B04’ and ‘Vi-BCZ14’ conditions respectively explain 9.7 %, 10 % and 16.3 % of the total variation while the second components explain 7.4 %, 5.5 % and 4.8 % of the total variation. Overlapping ellipses and similar component percentages tend to show comparable variability among the experimental designs.

## 4 Experimental Design, Materials and Methods

### 4.1 Biological material

All experiments were conducted on a pseudo-F1 population described in [3]. The 267 individuals were derived from a controlled cross between TN10-8 and ‘Fiesta’, two genotypes partially resistant to apple scab. Each year, scions were collected from a conservatory orchard located at the Institut National de Recherche pour l’Agriculture, l’Alimentation et l’Environnement (INRAE, Angers, France) and maintained by the Horticole Experimental Unit (10.15454/1.5573931618268674E12). Scions were grafted on MM106 rootstock.

Three experiments were conducted: one without pathogen and the others with inoculation of two distinct isolates of *Vi*. The first inoculation was realized with the reference isolate ‘EU-B04’ (Origin: Belgium, host: Golden Delicious) previously described in [9,10] whereas the second one, used the isolate ‘09BCZ014’ (Origin: France, host: ‘TN10-8 × Prima’ progeny [individual E063]), referred to as isolate ‘2557’ in [3]. The isolate ‘09BCZ014’ partially overcomes the resistance conferred by the ‘qT1’ resistance QTL segregating in the ‘TxF progeny’. The use of this isolate allows to better study the effect of other QTL segregating in the progeny.

Monoconidial suspensions were prepared from diseased dry leaves at a concentration of 2.5 × 10^5^ conidia.mL^−1^ and sprayed on grafted trees, incubated thereafter two days at 17 °C under a plastic sheet to maintain high humidity, according to the conditions described by [11].

### 4.2 Experimental design and sampling

After grafting, young trees were grown in a greenhouse at INRAE Angers (France) under semi-controlled conditions (23 °C day/20 °C night, humidity 40–80 %, and artificial light to complement natural light). Leaf sampling was performed on approximatively 5-weeks old trees, non-inoculated or five days after inoculation with *Vi*. All information related to the design and sampling of the three experiments is described in Table 1. Sample sizes vary depending on the experiment (see LIMITATIONS section for more details).

**Table 1:** Summary of information about the design and sampling strategies for the three experiments conducted on the ‘TxF progeny’.

Experiment	No inoculation	Vi-B04	Vi-BCZ14
Time and year	Spring 2021	Spring 2022	Autumn 2022
Number of genotypes	117	256	229
Number of replicated genotypes	23	256	108
Total number of samples	140	512	337
Number of copies per sample	1	Pool of 2	Pool of 2
Randomization (Block-design)	NO	YES	YES
Metabolic features detected	1,576	2,102	2,955

In the experiment of 2021 without inoculation (coded ‘No inoc.’), 117 genotypes out of the 267 individuals of the ‘TxF progeny’ were grafted in two replicates. The resulting plants were grown without randomization in the greenhouse. Out of the 117 genotypes, 94 were sampled as unique whereas 23 were duplicated. Two leaves were collected on each plant and the two halves of both leaves were pooled in an Eppendorf tube. A total of 140 samples were then processed for metabolomic analysis.

In *Vi*-experiments of 2022 (coded ’Vi-B04’ and ‘Vi-BCZ14’), 256 genotypes were grafted in eight replicates and distributed in four randomized blocks, containing each, two replicates of the same genotype placed side by side. Plants were inoculated with ‘EU-B04’ isolate in Spring 2022 and with isolate ‘09BCZ014’ in Autumn 2022. At the end of the first experiment, plants were treated with antifungal chemicals (Score® and Funguran®) and then pruned leading to a fewer number of genotypes and replicates for the second experiment. Sampling was done on two blocks containing the best growing shoots. For each block, one leaf was collected on each replicate and cut in half. Thus, a sample is composed of two half-leaves from the two replicates of the same block. Metabolomic analyses were performed on each of these samples resulting in a total of 512 samples for ‘Vi-B04’ experiment and 337 samples for ‘Vi-BCZ14’ experiment.

### 4.3 Extraction of metabolites and chemical profiling

For all three experiments, samples were directly frozen in liquid nitrogen and kept at -80 °C until lyophilization. Leaf samples were then grounded using a ball mills with two balls of 3–4 mm of diameter in each tube. Metabolites were extracted from 20 mg dry powder of apple leaves as described in [12]. The dry powder was extracted in 1 mL 60 % aqueous methanol to which 25 µL of taxifolin were added (as internal standard, 2 mg.mL^−1^ methanol), then blended (1 min) and centrifuged (10 min, 2800 × g). The extraction was repeated once, and the supernatants pooled and vacuum-dried. The residue was dissolved in 500 µL of 70 % aqueous methanol, filtered (0.22 µm) and transferred to vials before HRMS^1^ analyses. One additional vial, the pooled-sample vial, was prepared with an equal quantity of each sample (2 µL each) for the HRMS^2^ analyses.

Chromatographic analyses were performed on a Vanquish UHPLC system equipped with a binary pump, an autosampler and a temperature-controlled column. Metabolites contained in the extracts (10 µL) were separated on a XB-C18 Kinetex (150 × 2.1 mm, 2.6 µm) (Phenomenex Inc., Torrance, CA, USA) using a gradient of mobile phase composed of water + 0.1 % formic acid (A) and methanol + 0.1 % formic acid (B) at a flow rate of 200 µL.min^−1^. The elution program consisted in starting with 10 % B for 2 min, then linearly increasing from 10 to 30 % B in 8 min, then to 95 % B in 10 min. The column was rinsed for 5 min with 95 % B and re-equilibrated to the initial conditions for 4 min prior to the next run. The samples were analysed randomly.

HRMS^1^ detection was performed on an Orbitrap IDX^TM^ (ThermoFisher Scientific, Bremen, Germany) mass spectrometer in positive and negative electrospray ionization (ESI) modes. The capillary voltages were set at 3.5 kV and 2.5 kV for positive and negative modes, respectively. The source gases were set (in arbitrary unit min^−1^) to 40 (sheath gas), 8 (auxiliary gas) and 1 (sweep gas) and the vaporizer temperature was 320 °C. Full scan HRMS^1^ spectra were acquired from 120 to 1200 *m*/*z* at a resolution of 60,000 (at 200 *m*/*z*). HRMS^2^ analysis was performed on the pooled-sample vial using the data dependent acquisition (DDA) mode. For this analysis, the AcquireX data acquisition workflow developed by ThermoFisher was applied. Briefly, this workflow increases the number of HRMS^2^ acquisition, especially on low-intensity ions, through the creation of an inclusion list after a first injection of the sample and the establishment of a dynamic exclusion list occurring by the iterative sample analysis (involving 5–6 successive injections).

The raw UHPLC-HRMS files were uploaded on the Compound Discoverer 3.3.2.31 software (ThermoFisher Scientific, Bremen, Germany) for metabolomics analysis. Briefly, it includes peak detection, chromatogram alignment and peak grouping in features. The Detect Compounds node was used with 5 ppm mass tolerance, 200,000 minimum peak intensity, at least 8 scans per peak, peak detection S/N threshold 3, remove baseline false, gap ratio threshold 0.35 and max peak width 1. All details are given in the analytic workflow available in Supplementary File 1. Each feature corresponds to a specific *m*/*z* at a given retention time.

### 4.4 Data exploration and visualization

All descriptive analyses were performed on published datasets [13] using the software R version 4.3.1 [14]. PCA were generated with the *PCA* function (FactoMineR package, [15]) and plotted with *fviz_pca_ind* function (factoextra package, [16]).

## Limitations

Fewer genotypes were grafted and repeated in the non-inoculated experiment (2021) than in the other two experiments, but this experiment was not designed to be compared with the other two but primarily to develop the untargeted metabolomic approach. In addition, the number of genotypes and replicates studied varied from experiment to experiment due to plant growth constraints.

## Ethics Statement

The authors have read and followed the ethical requirements for publication in Data in Brief and confirm that the current work does not involve human subjects, animal experiments, or any data collected from social media platforms*.*

## Credit Author Statement

**Romane Lapous:** Data acquisition. Data curation. Formal analysis. Writing - original draft/review/editing. **Florent Magot:** Data acquisition. Data curation. Writing - review/editing. **Romain Larbat:** Data acquisition. Methodology. Writing - review/editing. **Caroline Denancé:** Data acquisition. **Christian Cattanéo:** Data acquisition. **Hélène Muranty:** Conceptualization. Methodology. Writing - review/editing. **Charles-Eric Durel:** Conceptualization. Funding acquisition. Methodology. Writing - review/editing. **Julie Ferreira de Carvalho:** Conceptualization. Methodology. Writing - original draft/review/editing.

## Data Availability

entrepot.recherche.data.gouv.frMetabolomic dataset of an apple progeny carrying resistance quantitative trait loci to apple scab without or after inoculation of the pathogen Venturia inaequalis (Original data).

## Appendix Supplementary materials

Image, application 1
